# Supplementary material for: Predicting visual function by interpreting a neuronal wiring diagram
Source: Nature. 2024 Oct 2;634(8032):113–23. doi: 10.1038/s41586-024-07953-5 (PMC11446822; doi:10.1038/s41586-024-07953-5)
Supplement: Supplementary file 6 — CRF and ERF predictions for individual TmY4 and TmY9 cells. Analogous to Supplementary Data 3, but for TmY target types. Shown are the top four monosynaptic pathways, the strongest pathway passing through each of the top ten intermediary types (ranking from Extended Data Fig. 7), and the trisynaptic pathway Tm1–TmY–Dm3–TmY (see the section entitled Prediction of spatial normalization). [file 41586_2024_7953_MOESM6_ESM.zip › DataS4/TmY9q/720575940630510444.pdf]

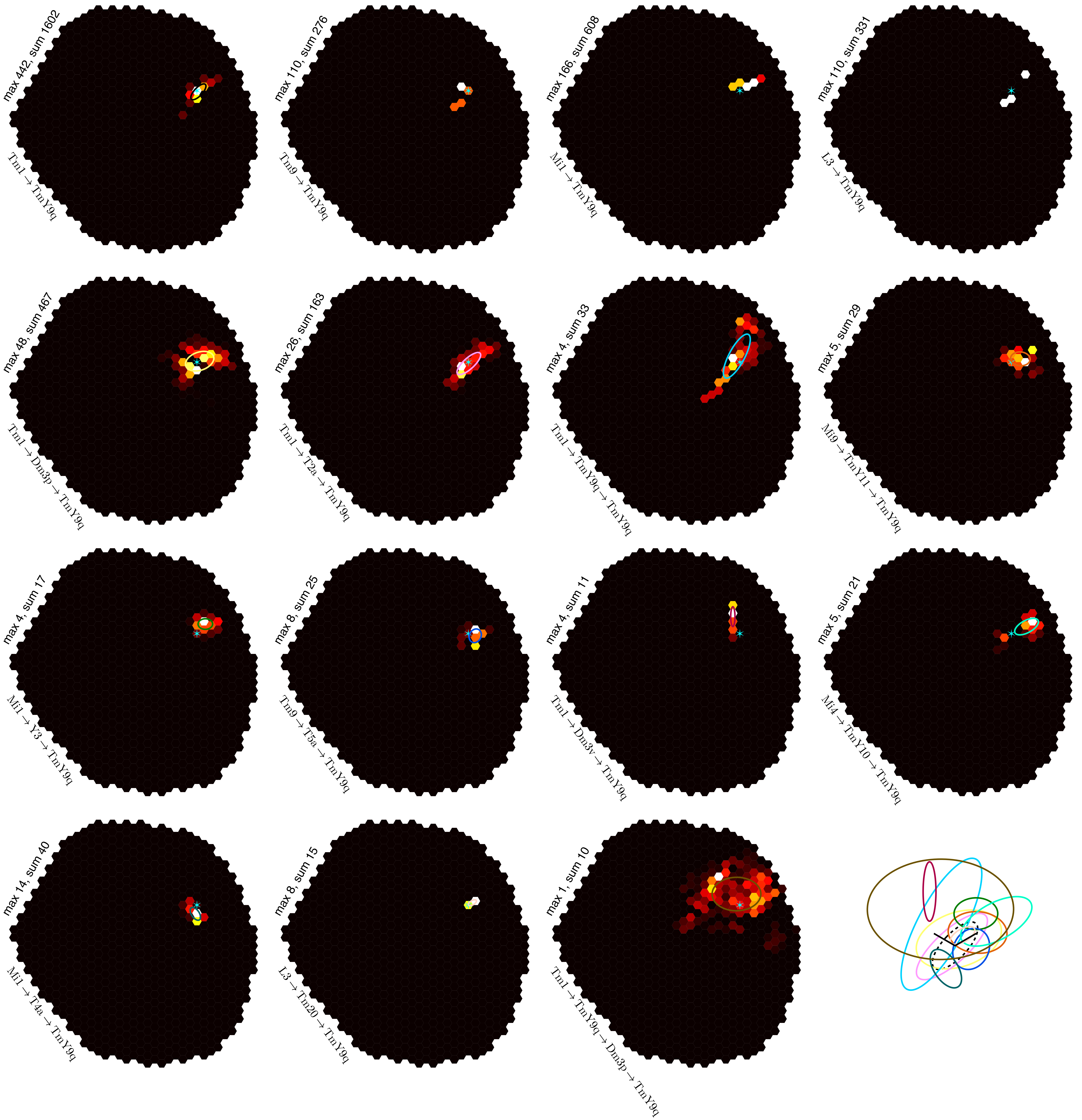
max 442, sum 1602
Tm1 → TmY9q
max 110, sum 276
Tm9 → TmY9q
max 166, sum 608
Mi1 → TmY9q
max 110, sum 331
L3 → TmY9q
max 48, sum 467
Tm1 → Dm3p → TmY9q
max 26, sum 163
Tm1 → T2a → TmY9q
max 4, sum 33
Tm1 → TmY9q → TmY9q
max 5, sum 29
Mi9 → TmY11 → TmY9q
max 4, sum 17
Mi1 → Y3 → TmY9q
max 8, sum 25
Tm9 → T5a → TmY9q
max 4, sum 11
Tm1 → Dm3v → TmY9q
max 5, sum 21
Mi4 → TmY10 → TmY9q
max 14, sum 40
Mi1 → T4a → TmY9q
max 8, sum 15
L3 → Tm20 → TmY9q
max 1, sum 10
Tm1 → TmY9q → Dm3p → TmY9q
